# Fasting blood glucose to high-density lipoprotein cholesterol ratio and MASLD risk: non-linear association and BMI mediation in non-diabetic adults

**DOI:** 10.3389/fnut.2026.1818931

**Published:** 2026-04-13

**Authors:** Yanyan Xuan, Dingting Wu, Qi Yao

**Affiliations:** 1 Department of Hospital Infection, The First Affiliated Hospital of Ningbo University, Ningbo, Zhejiang, China; 2 Department of Hepatology, The First Affiliated Hospital of Ningbo University, Ningbo, Zhejiang, China; 3 Department of Geriatrics Medicine, The First Affiliated Hospital of Ningbo University, Ningbo, Zhejiang, China; 4 Department of Clinical Nutrition, Sir Run Run Shaw Hospital, Zhejiang University School of Medicine, Hangzhou, Zhejiang, China

**Keywords:** body mass index, fasting blood glucose, high-density lipoprotein cholesterol, mediation analysis, metabolic dysfunction-associated steatotic liver disease, nonlinear relationship

## Abstract

**Background:**

The fasting blood glucose (FBG) to high-density lipoprotein cholesterol (HDL-C) ratio (GHR) integrates glucose and lipid metabolism, but its association with metabolic dysfunction-associated steatotic liver disease (MASLD) is unclear. We aimed to investigate the relationship between GHR and MASLD and to quantify the mediating role of body mass index (BMI) in non-diabetic adults.

**Methods:**

This cross-sectional study included 13,682 non-diabetic Japanese adults from the NAGALA cohort (2004–2015). Logistic regression was used to examine the association between GHR and MASLD risk, while generalized additive models (GAMs) and smooth curve fitting were used to investigate their non-linear relationship. Receiver operating characteristic (ROC) curve analysis was performed to evaluate the diagnostic efficacy of GHR for MASLD, and mediation analysis was conducted to assess the mediating effect of BMI in this association.

**Results:**

The prevalence of MASLD was 15.25% (*n* = 2,087). GHR was significantly higher in participants with MASLD (4.93 ± 1.14 vs. 3.59 ± 1.07). After multivariable adjustment, each 1-unit increase in GHR was associated with a 23% higher risk of MASLD (OR 1.23, 95% CI 1.15–1.31). A threshold effect was identified, with the risk escalating progressively when GHR < 4.62. GHR showed good discriminative ability with an AUC of 0.815 (95% CI: 0.806–0.824), significantly outperforming FBG alone (AUC 0.727) and HDL-C alone (AUC 0.787). Mediation analysis revealed that BMI accounted for 59.86% of the total association between GHR and MASLD risk as a potential intermediary factor.

**Conclusion:**

Among non-diabetic Japanese adults, elevated GHR is independently associated with an increased risk of MASLD, and BMI acts as a potential partial intermediary factor in this observed association. GHR may serve as a simple, cost-effective initial screening tool for MASLD in non-diabetic clinical practice, particularly in individuals without severe obesity or hypertriglyceridemia.

## 1 Introduction

Metabolic dysfunction-associated steatotic liver disease (MASLD) has emerged as the most prevalent chronic liver disease globally, imposing a substantial public health burden due to its increasing association with liver fibrosis, cirrhosis, and hepatocellular carcinoma (1–6). Over the past three decades, its global prevalence has overtaken that of chronic viral hepatitis and alcoholic liver disease—underscoring an urgent need for optimized screening and risk stratification strategies (6–9). Although liver biopsy remains the gold standard for diagnosing MASLD, its inherent invasiveness and considerable cost have limited its large-scale clinical application (1, 7, 10, 11). Meanwhile, abdominal ultrasonography and existing non-invasive biomarkers lack adequate sensitivity for detecting early-stage lesions—an essential step to improve prognosis and reduce disease burden (12, 13). Thus, the development of a simple, reliable, and non-invasive screening biomarker represents a critical unmet clinical need in the management of MASLD.

Systemic metabolic dysregulation, particularly imbalances in glucose and lipid homeostasis, constitutes the central pathophysiological underpinning of MASLD development and progression (7, 14, 15). Fasting blood glucose (FBG) and high-density lipoprotein cholesterol (HDL-C) are key biomarkers of metabolic disturbance, each independently associated with an elevated risk of MASLD (16–23). Notably, low HDL-C levels often coexist with elevated FBG, and their synergistic effects exacerbate metabolic derangements (24). However, most prior studies have analyzed these indicators in isolation in the context of MASLD, overlooking their combined effects and resulting in suboptimal predictive performance of individual markers (24). Thus, composite biomarkers integrating glucose and lipid metabolic parameters may more comprehensively capture the underlying metabolic dysregulation in MASLD and offer superior risk stratification value.

The fasting blood glucose-to-high-density lipoprotein cholesterol ratio (GHR) is a novel composite biomarker that reflects integrated glucose and lipid metabolic status and has well-documented predictive value for metabolic disorders, such as coronary heart disease and cholelithiasis (25–27). The biological relevance of GHR to MASLD resides in its capacity to capture the synergistic dysregulation of glucose and lipid metabolism—a key driver of hepatic steatosis. Despite this theoretical rationale, the specific association between GHR and MASLD, its diagnostic performance, and its applicability in non-diabetic populations remains insufficiently explored. Most existing relevant studies have focused on non-alcoholic fatty liver disease (NAFLD) rather than MASLD, the latter of which encompasses a more comprehensive metabolic definition—creating a critical research gap.

Body mass index (BMI) is a key contributor to MASLD and a critical mediator linking systemic metabolic dysregulation to hepatic steatosis (28–31). Elevated BMI can mediate metabolic abnormalities and directly promote hepatic injury, suggesting that it may serve as a bridging factor in the association between GHR and MASLD (32–34). We hypothesized that BMI plays an important mediating role in the GHR–MASLD relationship: GHR reflects glucose–lipid metabolic disturbance that indirectly modulates BMI, ultimately increasing the risk of MASLD. However, this hypothesis has not been validated in non-diabetic populations, and the specific mediating proportion of BMI in this pathway remains elusive.

This study focused on non-diabetic adults to minimize confounding by severe metabolic disorders, which may obscure the independent role of GHR, and to target the early stage of metabolic abnormalities, where screening and intervention hold the greatest clinical value. Using large-scale cross-sectional data from the NAFLD in the Gifu Area, Longitudinal Analysis (NAGALA) cohort, we aimed to (1) elucidate the association between GHR and MASLD risk, such as its potential non-linear characteristics; (2) assess the diagnostic performance of GHR for MASLD and compare it with that of individual FBG and HDL-C; and (3) quantify the mediating proportion of BMI in this association.

## 2 Materials and methods

### 2.1 Study design and population

This study is a secondary analysis of publicly available data from the NAGALA study—a population-based prospective cohort investigation of chronic metabolic and liver diseases conducted in Gifu Prefecture, central Japan. Gifu was selected as the study setting due to its demographic representativeness and economic stability, which collectively support the generalizability of the study results to the broader Japanese adult population. Established in 1994, the NAGALA study has continuously enrolled participants who undergo annual or biennial health screenings at Murakami Memorial Hospital. Its primary objective is to identify modifiable risk factors for type 2 diabetes mellitus (T2DM), MASLD, and related conditions, thereby informing evidence-based public health strategies. All data—such as anthropometric measurements, biochemical profiles, and lifestyle assessments—were collected by trained medical personnel using standardized and validated protocols, ensuring high data quality and reliability.

The NAGALA dataset utilized in this study is publicly accessible via the Dryad Digital Repository (https://doi.org/10.5061/dryad.8q0p192), curated by Professor Takuro Okamura. Dryad is a non-profit, open-access repository dedicated to facilitating data reuse in research and education, providing unrestricted access to datasets. The original study authors have transferred copyright to the repository, enabling exploratory secondary analyses without copyright infringement. This study strictly adheres to Dryad’s Terms of Service. The original NAGALA study was approved by the Ethics Committee of Murakami Memorial Hospital (approval number: MMH-IRB-1994-001), and all participants provided written informed consent for the use of their de-identified data in future research. In line with international ethical guidelines for secondary analyses of publicly available de-identified data (Declaration of Helsinki, 2013 revision), no additional Institutional Review Board (IRB) approval was required for this study.

**Figure 1:**
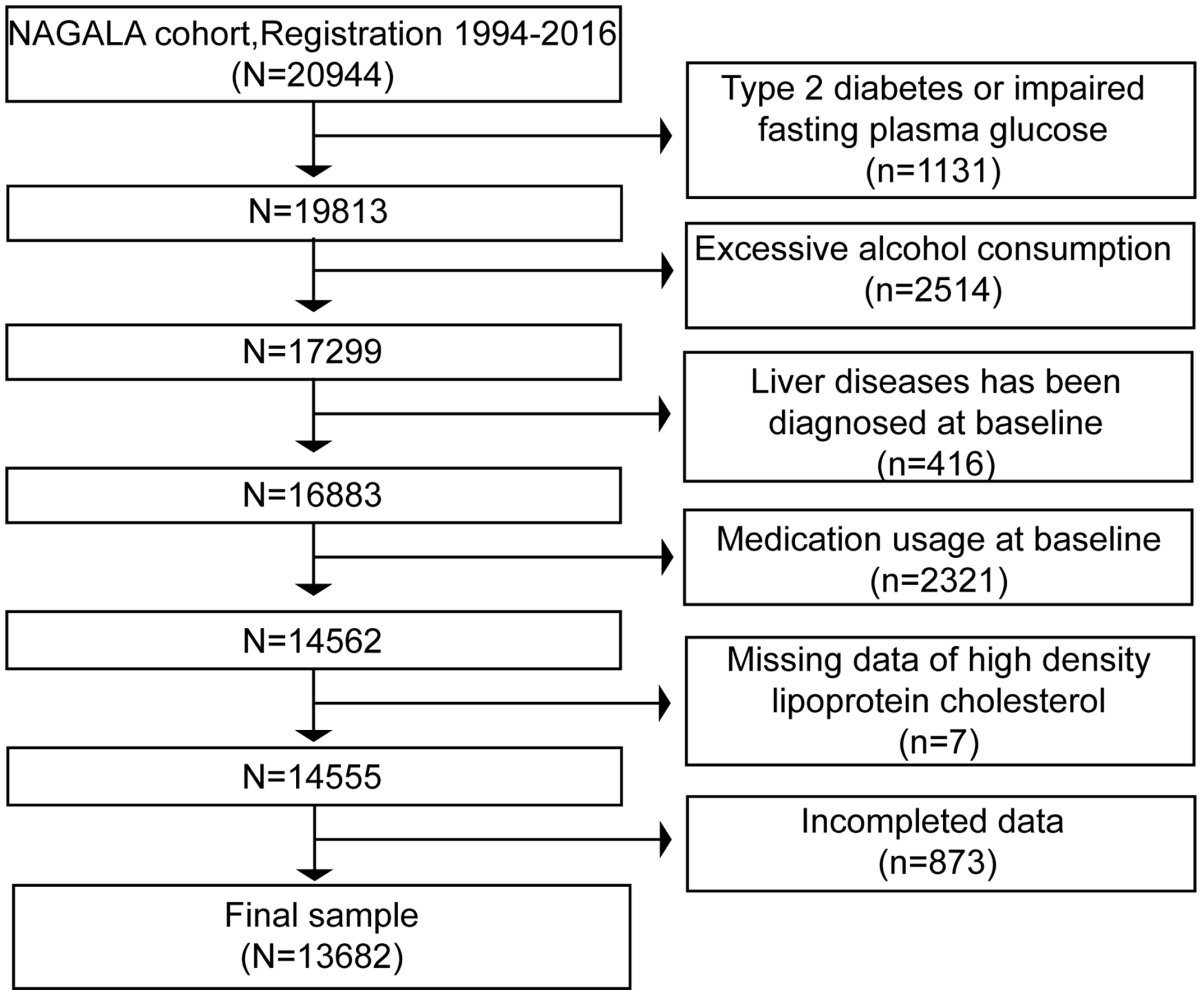
Flowchart of the study participants. NAGALA, nonalcoholic fatty liver disease in the Gifu area longitudinal analysis.

The initial study population comprised 20,944 Japanese adults who had undergone at least two health examinations at Murakami Memorial Hospital between January 2004 and December 2015. To enhance sample homogeneity and minimize confounding, participants were excluded according to the following criteria: (1) a prior diagnosis of T2DM (*n* = 323) or impaired fasting glucose (IFG), defined as fasting blood glucose (FBG) ≥ 6.1 mmol/L (*n* = 808); (2) excessive alcohol consumption, defined as daily ethanol intake > 30 g for men or > 20 g for women (*n* = 2,514); (3) a history of chronic liver disease, such as alcoholic liver disease and chronic viral hepatitis B/C, confirmed via serological testing or medical record review (*n* = 416); (4) regular use of medications potentially affecting glucose metabolism or hepatic steatosis, such as antihypertensives, lipid-lowering drugs, and glucocorticoids (*n* = 2,321); (5) missing key baseline data, such as anthropometric measurements, biochemical parameters, and lifestyle information (*n* = 863); and (6) incomplete HDL-C assay results (*n* = 7). After applying these exclusion criteria, 7,262 participants were excluded, yielding a final analytical sample of 13,682 non-diabetic Japanese adults. A participant flow diagram is provided in Figure 1.

### 2.2 Definition of MASLD

Hepatic steatosis, a core feature of MASLD, was diagnosed by board-certified sonographers using abdominal ultrasound. Sonographers were blinded to participants’ clinical data to minimize bias in the assessment. The ultrasound diagnosis was based on four validated imaging features consistent with international diagnostic standards for hepatic steatosis: (1) increased hepatic echogenicity relative to the renal cortex; (2) reduced liver and kidney echogenicity contrast; (3) blurring of intrahepatic vascular margins; and (4) deep acoustic attenuation in the hepatic parenchyma.

MASLD was defined according to the 2020 International Expert Consensus Statement (35), requiring the concurrent presence of both of the following criteria: (1) evidence of hepatic steatosis confirmed by histology, imaging (e.g., ultrasound), or validated blood-based biomarkers; and (2) at least one metabolic abnormality, as defined below: Overweight or obesity, defined as a BMI ≥ 23 kg/m^2^ (Asian-specific metabolic risk criteria); confirmed T2DM, based on the American Diabetes Association (ADA) criteria FBG ≥ 7.0 mmol/L, glycated hemoglobin (HbA1c) ≥ 6.5%, or physician-documented diagnosis; metabolic dysfunction, defined as the presence of ≥ 2 of the following: waist circumference (WC) ≥ 90 cm in men or ≥ 80 cm in women; blood pressure ≥ 130/85 mmHg or current use of antihypertensive medication; triglycerides (TG) ≥ 1.7 mmol/L or current use of lipid-lowering medication; HDL-C < 1.0 mmol/L in men or < 1.3 mmol/L in women or current use of lipid-lowering medication; and prediabetes (FBG 5.6–6.9 mmol/L or HbA1c 5.7–6.4%).

### 2.3 Variables

Comprehensive baseline data were extracted from the NAGALA database using standardized protocols to ensure consistency across all assessments. Trained medical staff administered a structured, validated questionnaire to collect demographic characteristics, anthropometric measures, lifestyle factors, and medical history. Venous blood samples were collected after an 8-h overnight fast and analyzed using a modular automated biochemical analyzer (Hitachi 7,600, Hitachi High-Technologies, Tokyo, Japan). All assays were performed and quality-controlled in accordance with Clinical and Laboratory Standards Institute (CLSI) guidelines to ensure the accuracy of biochemical measurements.

The primary exposure variable was the GHR, calculated as the ratio of FBG (mmol/L) to HDL-C (mmol/L). The primary outcome variable was MASLD status, defined as described in the Definition of MASLD section. Covariates were selected based on previous literature and clinical relevance. Continuous covariates included age, weight, systolic blood pressure (SBP), diastolic blood pressure (DBP), alcohol intake, total cholesterol (TC), low-density lipoprotein cholesterol (LDL-C), triglycerides (TG), aspartate aminotransferase (AST), alanine aminotransferase (ALT), *γ*-glutamyltransferase (GGT), HbA1c, and FBG. Categorical covariates included exercise habits (regular exercise: ≥ 1 session/week of moderate-to-vigorous physical activity; no regular exercise: <1 session/week), smoking status (never, former, or current), and alcohol consumption (never/light: < 40 g/week; moderate: 40–139 g/week; or heavy: ≥ 140 g/week) (36, 37). Hypertension was defined according to the Japanese Society of Hypertension Guidelines (JSH 2019) as SBP ≥ 140 mmHg and/or DBP ≥ 90 mmHg or current use of antihypertensive medication (38).

### 2.4 Statistical analysis

All statistical analyses were conducted using R software (Version 4.1.0; https://www.R-project.org) and EmpowerStats software (Version 4.0; https://www.empowerstats.com). A two-sided *p*-value < 0.05 was deemed statistically significant. Baseline characteristics were compared between participants with and without MASLD. The Shapiro–Wilk test was used to assess the normality of continuous variables, which were expressed as mean ± standard deviation (SD) for normally distributed data and median (interquartile range, IQR) for non-normally distributed data. Categorical variables were summarized as percentages. Intergroup comparisons were performed using independent-samples t-tests for normally distributed continuous variables, Mann–Whitney U tests for non-normally distributed continuous variables, and Pearson’s chi-square tests for categorical variables, respectively.

The GHR was categorized into quartiles (Q1: lowest; Q4: highest) based on the distribution of the study population, with Q1 designated as the reference group to explore potential relationships between GHR and MASLD. Multivariate logistic regression models were constructed to estimate odds ratios (ORs) and their corresponding 95% confidence intervals (CIs) for the association between GHR and MASLD risk. Three sequentially adjusted models were implemented: (1) Model 1 (unadjusted); (2) Model 2 (minimally adjusted), adjusting for age and sex; (3) Model 3 (fully adjusted), adjusting for age, sex, hypertension, smoking status, alcohol consumption status, exercise habits, HbA1c, ALT, BMI, AST, GGT, TG, and LDL-C. To verify the robustness of the primary findings, sensitivity analyses were performed via subgroup stratification by sex (men vs. women), age (< 40 vs. ≥ 40 years), BMI (< 25 vs. ≥ 25 kg/m^2^), and TG (< 1.7 vs. ≥ 1.7 mmol/L).

Generalized additive models (GAMs) were employed to explore potential non-linear associations between the GHR and MASLD risk. Based on the smoothing curves generated from GAMs, two-piece linear regression models were constructed to identify threshold values at which the strength or direction of the association between GHR and MASLD risk changed significantly. Receiver Operating Characteristic (ROC) curves were plotted to compare the diagnostic efficacy of GHR, FBG, and HDL-C for MASLD. The area under the ROC curve (AUC) was calculated for each indicator, and pairwise comparisons of AUCs were performed using the DeLong test to determine statistically significant differences in diagnostic performance between the three markers.

A three-step mediation analysis was performed using the mediation package in R to investigate whether BMI mediated the association between GHR and MASLD risk. The analysis proceeded as follows:(1) linear regression was used to evaluate the association between GHR and BMI, with adjustment for all covariates included in the fully adjusted model;(2) logistic regression was applied to examine the association between BMI and MASLD, adjusted for the same set of covariates; (3) bootstrap resampling with 1,000 iterations was conducted to estimate the total, direct, and indirect effects, as well as the mediation proportion, calculated as (indirect effect / total effect) × 100%. Mediation was deemed statistically significant if the 95% CI for the indirect effect excluded zero, accompanied by significant total and indirect effects (both *p* < 0.05) and a positive mediation proportion.

## 3 Results

### 3.1 Baseline demographic and clinical characteristics

A total of 13,682 participants were enrolled in this study, of whom 2,087 were diagnosed with MASLD, yielding a prevalence of 15.25%. The overall study population had a mean age of 43.35 ± 8.81 years, with 52.89% being men and 47.11% women. Detailed baseline characteristics stratified by MASLD status are summarized in Table 1. Compared with participants without MASLD, those with MASLD were more likely to be women, aged ≥ 40 years, and having a BMI ≥ 25 kg/m^2^. Regarding lifestyle factors and comorbidities, the MASLD group exhibited significantly higher rates of smoking (ever and current) and hypertension. In contrast, the proportion of participants with regular exercise habits was significantly lower in the MASLD group than in the non-MASLD group (*p* < 0.001). Biochemically and anthropometrically, participants with MASLD had significantly lower HDL-C levels. At the same time, significantly higher values were observed for BMI, body weight, WC, SBP, DBP, FBG, HbA1c, TC, LDL-C, ALT, AST, GGT, and TG (all *p* < 0.001). Additionally, the GHR was significantly higher in the MASLD group compared with the non-MASLD group (4.93 ± 1.14 vs. 3.59 ± 1.07, *p* < 0.001).

**Table 1:** Characteristics of participants with and without MASLD status.

Characteristics	Non- MASLD (*n* = 11,595)	MASLD (n = 2,087)	*p* value
Age (years)	43.16 ± 8.92	44.55 ± 8.31	< 0.001
Age (%)			< 0.001
< 40	38.84	31.34	
≥ 40	61.16	68.66	
Gender (%)			< 0.001
Men	56.72	18.64	
Women	43.28	81.36	
Smoking behavior (%)			< 0.001
Never smoke	66.21	46.62	
Ever smoke	15.74	25.01	
Current smoke	18.05	28.37	
Drinking behavior (%)			< 0.001
Never/light drink	86.02	87.59	
Moderate drink	12.93	12.22	
Heavy drink	1.05	0.19	
Hypertension (%)			< 0.001
No	96.45	84.48	
Yes	3.55	15.52	
Habit of exercise (%)			< 0.001
No	82.34	85.72	
Yes	17.66	14.28	
BMI (kg/m^2^)	21.28 ± 2.59	26.08 ± 2.96	< 0.001
BMI (%)			< 0.001
< 25	92.04	41.26	
≥ 25	7.96	58.74	
Weight (kg)	57.37 ± 9.85	73.80 ± 10.86	< 0.001
Height (cm)	163.81 ± 8.42	168.05 ± 7.89	< 0.001
WC (cm)	73.89 ± 7.84	87.15 ± 7.45	< 0.001
SBP (mmHg)	111.63 ± 13.91	124.54 ± 14.71	< 0.001
DBP (mmHg)	69.46 ± 9.73	78.56 ± 10.11	< 0.001
FBG (mmol/L)	5.09 ± 0.40	5.41 ± 0.36	< 0.001
HbA1c (%)	5.16 ± 0.31	5.32 ± 0.33	< 0.001
ALT (IU/L)	15.00 (12.00–20.00)	28.00 (21.00–40.00)	< 0.001
GGT (IU/L)	13.00 (11.00–18.00)	23.00 (17.00–33.00)	< 0.001
AST (IU/L)	17.00 (14.00–20.00)	21.00 (17.00–26.00)	< 0.001
TG (mmol/L)	0.64 (0.45–0.94)	1.30 (0.90–1.86)	< 0.001
TC (mmol/L)	5.06 ± 0.86	5.46 ± 0.86	< 0.001
HDL-C (mmol/L)	1.52 ± 0.40	1.15 ± 0.26	< 0.001
LDL-C (mmol/L)	3.19 ± 0.78	3.63 ± 0.80	< 0.001
GHR	3.59 ± 1.07	4.93 ± 1.14	< 0.001

Mean ± SD or medians (quartile interval) were for continuous variables. % was for categorical variables.

MASLD, metabolic dysfunction-associated steatotic liver disease; BMI, body mass index; WC, waist circumference; SBP, systolic blood pressure; DBP, diastolic blood pressure; ALT, alanine aminotransferase; AST, aspartate aminotransferase; GGT, gamma-glutamyl transpeptidase; HbA1c, glycosylated hemoglobin; FBG, fasting blood glucose; TG, triglycerides; TC, total cholesterol; HDL-C, high-density lipoprotein cholesterol; LDL-C, low-density lipoprotein cholesterol; GHR, fasting blood glucose to high-density lipoprotein cholesterol ratio.

### 3.2 Association between GHR and MASLD

Multivariate logistic regression analyses were performed to evaluate the association between the GHR and MASLD risk (Table 2). GHR was positively associated with MASLD risk across all three sequentially adjusted models (all *p* < 0.001). In Model 1 (unadjusted), the OR for each 1-unit increase in GHR was 2.52 (95% CI: 2.41–2.63). After adjusting for age and sex (Model 2), the OR was 2.17 (95% CI: 2.07–2.28), and in the fully adjusted Model 3 (adjusted for all covariates listed in the Methods section), the OR was 1.23 (95% CI: 1.15–1.31). When GHR was categorized into quartiles (Q1: 1.35–2.91, Q2: 2.91–3.60, Q3: 3.60–4.51, Q4: 4.51–11.10) with Q1 as the reference group, a significant positive relationship was observed across all models (all P for trend < 0.001). In the fully adjusted Model 3, the ORs for MASLD risk in Q2, Q3, and Q4 were 1.62, 2.31, and 3.29, respectively (all *p* < 0.001).

**Table 2:** Associations between GHR and the MASLD risk.

	Model 1 OR (95% CI), *p* value	Model 2 OR (95% CI), *p* value	Model 3 OR (95% CI), *p* value
GHR	2.52 (2.41, 2.63) < 0.001	2.17 (2.07, 2.28) < 0.001	1.23 (1.15, 1.31) < 0.001
Q1 (1.35–2.91)	Reference	Reference	Reference
Q2 (2.91–3.60)	3.72 (2.71, 5.11) < 0.001	3.16 (2.30, 4.35) < 0.001	1.62 (1.13, 2.31) < 0.001
Q3 (3.60–4.51)	12.92 (9.63, 17.33) < 0.001	9.09 (6.74, 12.27) < 0.001	2.31 (1.64, 3.25) < 0.001
Q4 (4.51–11.10)	41.73 (31.31, 55.64) < 0.001	25.18 (18.69, 33.94) < 0.001	3.29 (2.33, 4.64) < 0.001
*P* for trend	< 0.001	< 0.001	< 0.001
Subgroup analysis			
Sex			
Men	2.85 (2.57, 3.17) < 0.001	2.75 (2.47, 3.05) < 0.001	1.20 (1.03, 1.40) 0.021
Women	2.03 (1.92, 2.14) < 0.001	2.03 (1.92, 2.14) < 0.001	1.23 (1.14, 1.32) < 0.001
Age (years)			
< 40	2.81 (2.59, 3.05) < 0.001	2.34 (2.14, 2.56) < 0.001	1.18 (1.03, 1.35) < 0.001
≥ 40	2.39 (2.26, 2.52) < 0.001	2.10 (1.98, 2.22) < 0.001	1.25 (1.16, 1.36) < 0.001
BMI (kg/m^2^)			
< 25	2.41 (2.27, 2.56) < 0.001	2.00 (1.87, 2.13) < 0.001	1.47 (1.36, 1.60) < 0.001
≥ 25	1.62 (1.49, 1.76) < 0.001	1.46 (1.34, 1.60) < 0.001	1.17 (1.05, 1.30) < 0.001
TG (mmol/L)			
< 1.7	2.44 (2.31, 2.57) < 0.001	2.09 (1.97, 2.21) < 0.001	1.22 (1.13, 1.32) < 0.001
≥ 1.7	1.38 (1.25, 1.53) < 0.001	1.35 (1.21, 1.50) < 0.001	1.16 (1.01, 1.33) < 0.001

Model 1: No covariates were adjusted. Model 2: age and gender were adjusted. Model 3: age, gender, hypertension, smoking status, drinking status, exercise status, HbA1c, BMI, ALT, AST, GGT, TG, and LDL-C were adjusted. In the subgroup analysis for gender, the model was not adjusted for gender. In the subgroup analysis for age, the model was not adjusted for age. In the subgroup analysis for BMI, the model was not adjusted for BMI. In the subgroup analysis for TG, the model was not adjusted for TG.

MASLD, metabolic dysfunction-associated steatotic liver disease; BMI, body mass index; WC, waist circumference; SBP, systolic blood pressure; DBP, diastolic blood pressure; ALT, alanine aminotransferase; AST, aspartate aminotransferase; GGT, gamma-glutamyl transpeptidase; HbA1c, glycosylated hemoglobin; FBG, fasting blood glucose; TG, triglycerides; TC, total cholesterol; HDL-C, high-density lipoprotein cholesterol; LDL-C, low-density lipoprotein cholesterol; GHR, fasting blood glucose to high-density lipoprotein cholesterol ratio.

Subgroup analyses were conducted by sex, age, BMI, and TG, with adjustments consistent with the fully adjusted Model 3 for each subgroup. The positive association between GHR and MASLD risk remained statistically significant across all subgroups (all *p* < 0.001). For sex stratification, each 1-unit increase in GHR was positively associated with MASLD risk across all three models in both women and men: women (Model 1: OR = 2.03, 95% CI: 1.92–2.14; Model 2: OR = 2.03, 95% CI: 1.92–2.14; Model 3: OR = 1.23, 95% CI: 1.14–1.32) and men (Model 1: OR = 2.85, 95% CI: 2.57–3.17; Model 2: OR = 2.75, 95% CI: 2.47–3.05; Model 3: OR = 1.20, 95% CI: 1.03–1.40). Regarding age stratification, the positive association was consistent across all models in participants aged < 40 years (Model 1: OR = 2.81, 95% CI: 2.59–3.05; Model 2: OR = 2.34, 95% CI: 2.14–2.56; Model 3: OR = 1.18, 95% CI: 1.03–1.35), with a similarly significant positive association observed in those aged ≥ 40 years. Similarly, significant positive associations were consistently detected in participants with BMI < 25 or ≥ 25 kg/m^2^, as well as in those with TG < 1.7 or ≥ 1.7 mmol/L (all *p* values < 0.001).

### 3.3 Association between GHR and BMI

As shown in Table 3, regression analyses confirmed a robust positive association between BMI and GHR (all *p* < 0.001). Specifically, for Model 1 (unadjusted): *β* = 1.24 (95% CI: 1.20–1.28); for Model 2 (adjusted for age and sex): *β* = 1.07 (95% CI: 1.03–1.12); and for Model 3 (fully adjusted): *β* = 0.70 (95% CI: 0.65–0.75). Additionally, GHR showed a significant positive relationship with increasing BMI quartiles (Q1: lowest, reference), with *p* value for trend < 0.001. Compared with Q1, the adjusted mean differences (β) for Q2, Q3, and Q4 were 0.83 (95% CI: 0.70–0.95), 1.47 (95% CI: 1.34–1.60), and 2.22 (95% CI: 2.07–2.38), respectively.

**Table 3:** Association between GHR and BMI.

Variables	Model 1 β (95% CI), *p* value	Model 2 β (95% CI), *p* value	Model 3 β (95% CI), *p* value
GHR	1.24 (1.20, 1.28) < 0.001	1.07 (1.03, 1.12) < 0.001	0.70 (0.65, 0.75) < 0.001
GHR quartile			
Q1 (1.35–2.91)	Reference	Reference	Reference
Q2 (2.91–3.60)	1.11 (0.98, 1.24) < 0.001	0.97 (0.83, 1.10) < 0.0001	0.83 (0.70, 0.95) < 0.0001
Q3 (3.60–4.51)	2.36 (2.22, 2.49) < 0.001	2.01 (1.87, 2.15) < 0.0001	1.47 (1.34, 1.60) < 0.0001
Q4 (4.51–11.10)	3.92 (3.79, 4.05) < 0.001	3.39 (3.24, 3.54) < 0.0001	2.22 (2.07, 2.38) < 0.0001
*P* for trend	< 0.001	< 0.001	< 0.001

Model 1: No covariates were adjusted. Model 2: age and gender were adjusted. Model 3: age, gender, hypertension, smoking status, drinking status, exercise status, HbA1c, ALT, AST, GGT, TG, and LDL-C were adjusted.

GHR, fasting blood glucose to high-density lipoprotein cholesterol ratio; BMI, body mass index; HbA1c, glycosylated hemoglobin; ALT, alanine aminotransferase; AST, aspartate aminotransferase; GGT, gamma-glutamyl transpeptidase; TG, triglycerides; LDL-C, low-density lipoprotein cholesterol; β, standardized regression coefficient; CI, confidence interval.

### 3.4 Relationship between BMI and MASLD

To evaluate the association between BMI and the risk of MASLD, a multivariate logistic regression analysis was conducted; the results are summarized in Table 4. BMI exhibited a consistent positive association with MASLD risk across all three regression models. In Model 1, each one-unit increase in BMI was associated with a 1.81-fold higher risk of MASLD (OR = 1.81, 95% CI: 1.77–1.86). After adjusting for potential confounders, age, and gender (Model 2), the association remained robust, with an OR of 1.79 (95% CI: 1.74–1.83). In the fully adjusted Model 3, the OR was 1.62 (95% CI: 1.57–1.66), confirming that BMI is an independent risk factor for MASLD.

**Table 4:** Association between BMI and the MASLD risk.

Variables	Model 1 OR (95% CI), *p* value	Model 2 OR (95% CI), *p* value	Model 3 OR (95% CI), *p* value
BMI (kg/m^2^)	1.81 (1.77, 1.86) < 0.001	1.79 (1.74, 1.83) < 0.001	1.62 (1.57, 1.66) < 0.001
BMI quartile (kg/m^2^)			
Q1 (13.77–19.78)	Reference	Reference	Reference
Q2 (19.78–21.66)	17.59 (5.49, 56.37) <0.001	13.84 (4.32, 44.37) < 0.001	12.40 (3.65, 42.13) < 0.001
Q3 (21.66–23.78)	147.50 (47.33, 459.69) < 0.001	98.05 (31.43, 305.89) < 0.001	66.59 (20.15, 220.09) < 0.001
Q4 (23.78–49.92)	1049.13 (337.60, 3260.31) < 0.001	681.51 (219.14, 2119.47) < 0.001	329.38 (100.01, 1084.77) < 0.001
*P* for trend	< 0.001	< 0.001	< 0.001

Model 1: No covariates were adjusted. Model 2: age and gender were adjusted. Model 3: age, gender, hypertension, smoking status, drinking status, exercise status, HbA1c, ALT, AST, GGT, TG, and LDL-C were adjusted.

MASLD, metabolic dysfunction-associated steatotic liver disease; BMI, body mass index; HbA1c, glycosylated hemoglobin; ALT, alanine aminotransferase; AST, aspartate aminotransferase; GGT, gamma-glutamyl transpeptidase; TG, triglycerides; LDL-C, low-density lipoprotein cholesterol; OR, odds ratios; CI, confidence interval.

When BMI was stratified into quartiles (Q1: 13.77–19.78 kg/m^2^, Q2: 19.78–21.66 kg/m^2^, Q3: 21.66–23.78 kg/m^2^, Q4: 23.78–49.92 kg/m^2^), with Q1 as the reference group, a significant relationship was observed between ascending BMI quartiles and elevated MASLD risk (all *p values* for trend < 0.001). Specifically, in the fully adjusted Model 3, compared with Q1, the ORs for MASLD were 12.40 (95% CI: 3.65–42.13) in Q2, 66.59 (95% CI: 20.15–220.09) in Q3, and 329.38 (95% CI: 100.01–1084.77) in Q4.

### 3.5 Nonlinear relationship analysis

**Figure 2:**
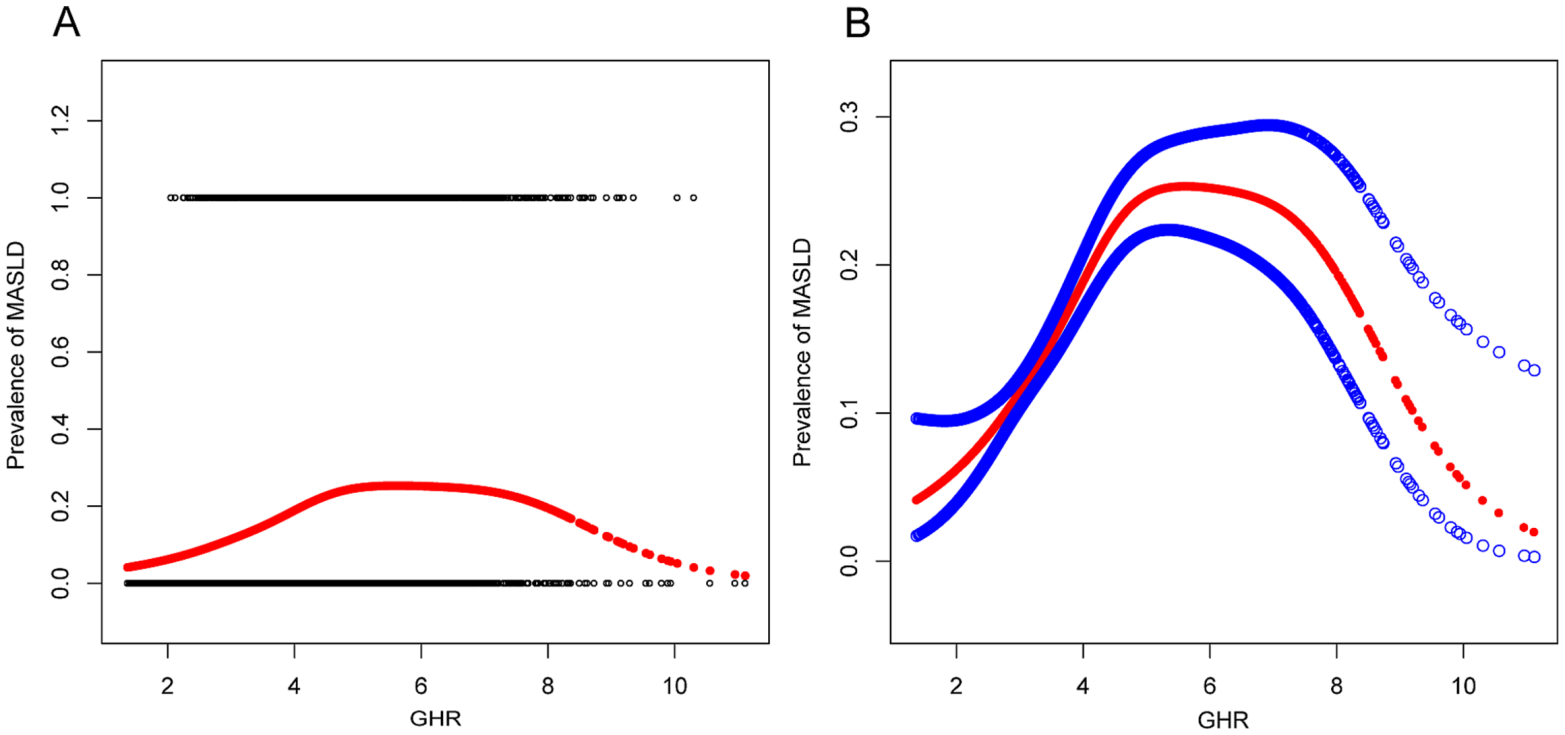
Associations between GHR and prevalence of MASLD. **(A,B)** Associations between GHR and prevalence of MASLD. The solid red line represents the smooth curve fit between variables. Blue bands represent the 95% confidence interval from the fit. They were adjusted for age, gender, hypertension, smoking status, drinking status, exercise status, HbA1c, BMI, ALT, AST, GGT, TG, and LDL-C. MASLD, metabolic dysfunction-associated steatotic liver disease; GHR, fasting blood glucose to high-density lipoprotein cholesterol ratio; BMI, body mass index; HbA1c, glycosylated hemoglobin; ALT, alanine aminotransferase; AST, aspartate aminotransferase; GGT, gamma-glutamyl transpeptidase; TG, triglycerides; LDL-C, low-density lipoprotein cholesterol.

**Figure 3:**
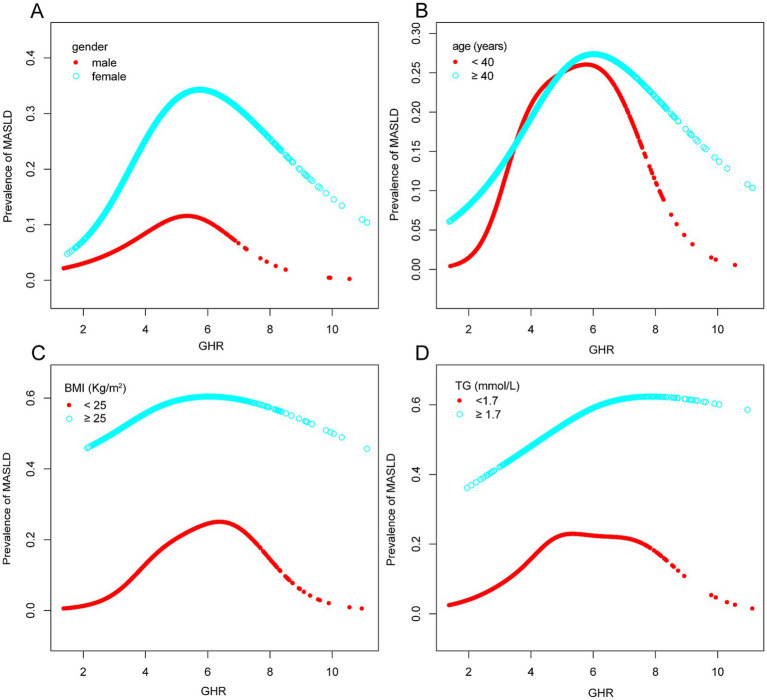
Associations between GHR and the prevalence of MASLD by gender **(A)**, age **(B)**, BMI **(C)**, and TG **(D)**. They were adjusted for age, gender, hypertension, smoking status, drinking status, exercise status, HbA1c, BMI, ALT, AST, GGT, TG, and LDL-C. In subgroup analyses, the model was not adjusted for the classified variables. MASLD, metabolic dysfunction-associated steatotic liver disease; GHR, fasting blood glucose to high-density lipoprotein cholesterol ratio; BMI, body mass index; HbA1c, glycosylated hemoglobin; ALT, alanine aminotransferase; AST, aspartate aminotransferase; GGT, gamma-glutamyl transpeptidase; TG, triglycerides; LDL-C, low-density lipoprotein cholesterol.

In this study, smooth curve fitting was applied to investigate the potential non-linear relationship between GHR and MASLD. A non-linear association between GHR and MASLD risk was identified in the overall population (Figure 2). Subgroup analyses stratified by age, sex, BMI, and TG levels consistently demonstrated a stable non-linear pattern across all subgroups (Figure 3). As shown in Table 5, threshold effect analysis using a two-piecewise linear regression model identified the inflection points of GHR as follows: 4.62 in the overall population; 4.61 in women; 4.73 in participants aged ≥ 40 years; 5.00 in those with BMI ≥ 25 kg/m^2^; and 6.40 in individuals with TG ≥ 1.7 mmol/L. On the left side of each inflection point, each unit increase in GHR was significantly associated with an elevated risk of MASLD (all *p* < 0.001). In contrast, on the right side of the inflection points, the associations were no longer statistically significant.

**Table 5:** Threshold effect analysis of GHR on the prevalence of MASLD in different genders, age, BMI, and TG using the two-piecewise linear regression model.

Prevalence of MASLD	Adjusted OR (95% CI), *p* value
All participants	
Fitting by the standard linear model	1.23 (1.15, 1.31), < 0.001
Fitting by the two-piecewise linear model	
Inflection point	4.62
GHR < 4.62	1.86 (1.62, 2.13), < 0.001
GHR > 4.62	0.92 (0.83, 1.02), 0.122
Log likelihood ratio	< 0.001
Women	
Fitting by the standard linear model	1.23 (1.14, 1.32), < 0.001
Fitting by the two-piecewise linear model	
Inflection point	4.61
GHR < 4.61	2.05 (1.71, 2.47), < 0.001
GHR > 4.61	0.94 (0.84, 1.05), 0.275
Log likelihood ratio	< 0.001
Age ≥ 40 (years)	
Fitting by the standard linear model	1.25 (1.16, 1.36), < 0.001
Fitting by the two-piecewise linear model	
Inflection point	4.73
GHR < 4.73	1.80 (1.55, 2.09), < 0.001
GHR > 4.73	0.94 (0.83, 1.07), 0.351
Log likelihood ratio	< 0.001
BMI ≥ 25 (kg/m^2^)	
Fitting by the standard linear model	1.17 (1.05, 1.30), 0.004
Fitting by the two-piecewise linear model	
Inflection point	5.00
GHR < 5.00	1.46 (1.22, 1.74), < 0.001
GHR > 5.00	0.91 (0.76, 1.10), 0.337
Log likelihood ratio	0.002
TG ≥ 1.7 (mmol/L)	
Fitting by the standard linear model	1.20 (1.05, 1.37), 0.006
Fitting by the two-piecewise linear model	
Inflection point	6.40
GHR < 6.40	1.39 (1.16, 1.66), < 0.001
GHR > 6.40	0.84 (0.59, 1.18), 0.309
Log likelihood ratio	0.025

Age, gender, hypertension, smoking status, drinking status, exercise status, HbA1c, BMI, ALT, AST, GGT, TG, and LDL-C were adjusted. In the subgroup analysis for gender, the model was not adjusted for gender. In the subgroup analysis for age, the model was not adjusted for age. In the subgroup analysis for BMI, the model was not adjusted for BMI. In the subgroup analysis for TG, the model was not adjusted for TG.

MASLD, metabolic dysfunction-associated steatotic liver disease; GHR, fasting blood glucose to high-density lipoprotein cholesterol ratio; BMI, body mass index; HbA1c, glycosylated hemoglobin; ALT, alanine aminotransferase; AST, aspartate aminotransferase; GGT, gamma-glutamyl transpeptidase; TG, triglycerides; LDL-C, low-density lipoprotein cholesterol; OR, odds ratios; CI, confidence interval.

### 3.6 A ROC analysis of GHR as a MASLD predictor

**Figure 4:**
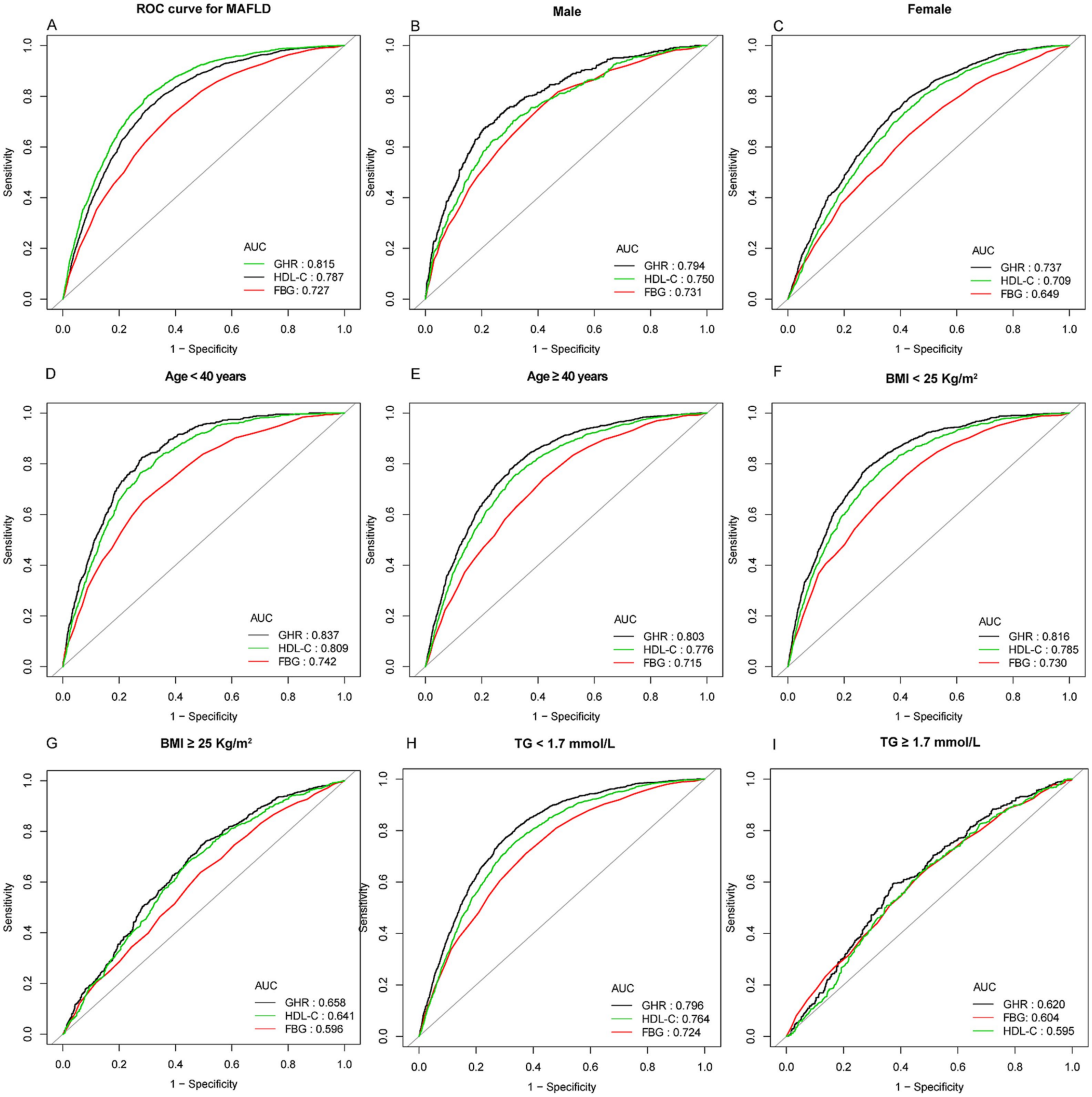
ROC curves comparing the predictive value of GHR with FBG and HDL-C for MASLD onset, with predictive efficacy assessed by AUC. **(A)** General population: GHR had superior predictive value for MASLD onset versus FBG and HDL-C. **(B,C)** Gender stratification: GHR outperformed FBG and HDL-C in men **(B)** and females **(C)**. **(D,E)** Age stratification: GHR showed better predictive performance in subjects < 40 years **(D)** and ≥ 40 years **(E)**. **(F,G)** BMI stratification: GHR showed higher predictive efficacy in BMI < 25 kg/m^2^ **(F)** and ≥ 25 kg/m^2^ **(G)**. (H, I) TG stratification: GHR outperformed FBG and HDL-C in TG < 1.7 mmol/L **(H)** and ≥1.7 mmol/L **(I)**. MASLD, metabolic dysfunction-associated steatotic liver disease; GHR, fasting blood glucose to high-density lipoprotein cholesterol ratio; BMI, body mass index; TG, triglycerides; FBG, fasting blood glucose; HDL-C, high-density lipoprotein cholesterol; ROC, receiver operating characteristic; AUC, area under the curve.

ROC curve analysis was performed to assess the predictive performance of GHR for MASLD, with FBG and HDL-C as comparators (Figure 4; Supplementary Table 1). In the overall study population, GHR yielded a significantly higher AUC of 0.8149 (95% CI: 0.8059–0.8239) compared with FBG (AUC 0.7267) and HDL-C (AUC 0.7866) (both *p* < 0.01). The optimal threshold for GHR in MASLD screening was determined to be 3.9735, with a specificity of 0.6980 and sensitivity of 0.8021 (Supplementary Table 1).

Stratified analyses by sex demonstrated that GHR maintained superior predictive performance for MASLD over FBG and HDL-C in both genders. In men, GHR achieved an AUC of 0.7942 (95% CI: 0.7715–0.8168) with an optimal cutoff of 3.6903 (specificity: 0.7922, sensitivity: 0.6735), whereas FBG and HDL-C exhibited lower AUCs (0.7311 and 0.7503, respectively). In women, GHR yielded an AUC of 0.7367 (95% CI: 0.7239–0.7496) with an optimal threshold of 4.4044 (specificity: 0.6289, sensitivity: 0.7373), compared with markedly lower AUCs for FBG (0.6489) and HDL-C (0.7094). Further subgroup analyses stratified by age, BMI, and TG levels consistently confirmed GHR’s superior predictive ability relative to FBG and HDL-C (Figure 4). For age subgroups, GHR exhibited an AUC of 0.8367 (95% CI: 0.8227–0.8507) in participants aged < 40 years (optimal cutoff: 3.9859, specificity: 0.7200, and sensitivity: 0.8242) and 0.8031 (95% CI: 0.7916–0.8146) in those aged ≥ 40 years (optimal cutoff: 4.0234, specificity: 0.6990, and sensitivity: 0.7788). For BMI subgroups, GHR yielded an AUC of 0.8156 (95% CI: 0.8023–0.8289) in individuals with BMI < 25 kg/m^2^ (optimal cutoff: 3.9619, specificity: 0.7195, and sensitivity: 0.7828) and 0.6575 (95% CI: 0.6341–0.6809) in those with BMI ≥ 25 kg/m^2^ (optimal cutoff: 4.2489, specificity: 0.5103, and sensitivity: 0.7447). For TG subgroups, GHR achieved an AUC of 0.7963 (95% CI: 0.7854–0.8073) in participants with TG < 1.7 mmol/L (optimal cutoff: 3.8908, specificity: 0.6969, and sensitivity: 0.7729) and 0.6197 (95% CI: 0.5871–0.6523) in those with TG ≥ 1.7 mmol/L (optimal cutoff: 5.2919, specificity: 0.6284, sensitivity: 0.5925). In all these stratified analyses, FBG and HDL-C consistently displayed inferior predictive performance compared with GHR. However, it is noteworthy that the predictive performance of GHR was suboptimal in specific subgroups. In participants with BMI ≥ 25 kg/m^2^, the AUC was 0.6575, and in those with TG ≥ 1.7 mmol/L, the AUC was 0.6197, suggesting limited diagnostic accuracy in these populations with advanced metabolic burden.

### 3.7 The mediating role of BMI in the relationship between GHR and MASLD

**Figure 5:**
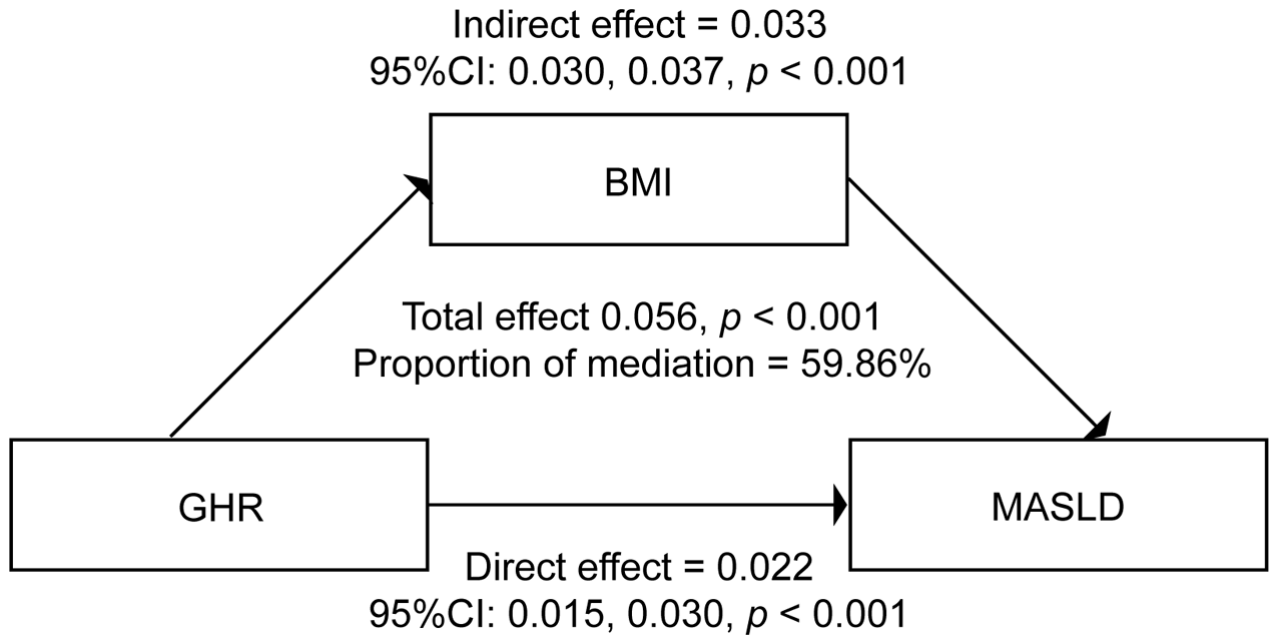
The mediation effect of BMI between GHR and MASLD. Adjusted for age, gender, hypertension, smoking status, drinking status, exercise status, HbA1c, ALT, AST, GGT, TG, and LDL-C. MASLD, metabolic dysfunction-associated steatotic liver disease; GHR, fasting blood glucose to high-density lipoprotein cholesterol ratio; BMI, body mass index; HbA1c, glycosylated hemoglobin; ALT, alanine aminotransferase; AST, aspartate aminotransferase; GGT, gamma-glutamyl transpeptidase; TG, triglycerides; LDL-C, low-density lipoprotein cholesterol.

Given the observed interrelationships among GHR, BMI, and MASLD, a mediation analysis was further performed to delineate the underlying mechanistic pathway linking these variables. In this model, GHR was designated as the independent variable, BMI as the mediating variable, and MASLD as the dependent variable. All analyses were adjusted for a comprehensive set of potential confounders, such as age, gender, hypertension, smoking status, drinking status, physical activity, HbA1c, ALT, AST, GGT, TG, and LDL-C. The mediation model structure and corresponding path coefficients are illustrated in Figure 5 and fully reported in Supplementary Table 2. Our results indicated that BMI acts as a potential partial intermediary factor in the association between GHR and MASLD. The total effect of GHR on MASLD risk was 0.056 (95% CI: 0.048–0.064; *p* < 0.001). The indirect effect of GHR on MASLD via BMI was 0.033 (95% CI: 0.030–0.037; *p* < 0.001), whereas the direct effect of GHR on MASLD remained statistically significant at 0.022 (95% CI: 0.015–0.030; *p* < 0.001) after accounting for the mediating pathway. The proportion of the total association between GHR and MASLD accounted for by BMI as a potential intermediary factor was 59.86% (*p* < 0.001).

## 4 Discussion

This cross-sectional study explored the association between GHR and MASLD risk, together with the potential mediating effect of BMI in this pathway. The overall prevalence of MASLD in the study population was 15.25%, and GHR levels were significantly higher in participants with MASLD than in those without. After multivariable adjustment, each 1-unit increment in GHR was associated with a 23% increased risk of MASLD, and subgroup analyses supported the consistency and robustness of this association across all subgroups examined.

A notable novel finding was a non-linear association between GHR and MASLD risk, with an inflection point at GHR = 4.62 in the overall population. These results indicate that MASLD risk rises progressively and significantly with increasing GHR up to this threshold, with no further significant association observed at GHR values above this inflection point. Several mechanisms may underlie this saturation pattern: first, the association between circulating GHR and hepatic fat accumulation or injury may weaken once GHR exceeds a threshold; second, individuals with extremely high GHR may exhibit greater metabolic heterogeneity; and third, unmeasured genetic or environmental confounders may partially contribute to this phenomenon. Nonetheless, the precise biological mechanisms underlying this non-linear pattern warrant further validation in prospective studies.

In addition, ROC curve analysis showed that GHR exhibited better discriminatory performance for MASLD than FBG or HDL-C alone. While GHR demonstrated superior overall performance, our subgroup analyses revealed that its predictive efficacy varied significantly. The notably lower AUCs observed in individuals with high BMI (≥25 kg/m^2^) or high TG (≥1.7 mmol/L) suggest that GHR may not fully capture the complexity of hepatic steatosis in the context of severe obesity or pronounced dyslipidemia. Therefore, while GHR is a valuable first-line screening metric, clinicians should exercise caution when applying it to patients with significant adiposity or mixed hyperlipidemia, where more comprehensive assessments may be warranted.

Furthermore, given that GHR integrates markers of both glucotoxicity and dyslipidemia, it may represent a broader biomarker for systemic cardiometabolic risk. Previous studies have validated the predictive value of GHR for various metabolic complications. For instance, research has shown that elevated GHR is associated with an increased risk of coronary heart disease, cholelithiasis, and even adverse outcomes in acute coronary syndrome patients. This suggests that a high GHR may act as a warning signal for clinicians. When identifying patients at risk for MASLD using GHR, physicians should also be vigilant for concomitant cardiovascular risks or early-stage glucose metabolism disorders, advocating for a more holistic approach to patient management.

Mediation analysis further revealed that BMI acts as a potential partial intermediary factor in the association between GHR and MASLD, with 59.86% of the total association accounted for by BMI, which provides preliminary insights into the potential relationship among these variables. To our knowledge, the present study is the first to quantify the proportion of the association between GHR and MASLD accounted for by BMI as a potential intermediary factor. These findings not only underscore the central role of BMI in the development of MASLD but also provide new insights into the metabolic mechanisms linking GHR to hepatic steatosis.

GHR is a newly proposed composite biomarker that integrates circulating lipid and glucose levels. Accumulating evidence has supported its potential as a predictive indicator for various diseases, playing a valuable role in assessing disease risk and prognosis in conditions, such as acute coronary syndrome, cholelithiasis, acute heart failure, and abdominal aortic calcification (25–27, 39, 40). In recent years, Guo et al. first validated the clinical utility of GHR in a retrospective study involving 6,645 Chinese non-diabetic patients with acute coronary syndrome who underwent percutaneous coronary intervention (40). Their results showed that a GHR ≥ 6.30 was an independent predictor of adverse outcomes, with elevated GHR associated with a 1.28-fold increased risk of long-term cardiovascular events. More recently, Wu et al. conducted a cross-sectional study of 3,898 American adults and identified a significant positive correlation between GHR and cholelithiasis prevalence; the risk of cholelithiasis in the high-GHR group was twice that in the low-GHR group (27).

While the association of GHR with cardiometabolic and biliary tract diseases is well established, evidence regarding its relationship with MASLD remains extremely limited. Notably, these aforementioned diseases and MASLD share core pathophysiological pathways, such as insulin resistance (IR), oxidative stress, and chronic inflammation (41–44). This shared mechanistic basis suggests that combining GHR with other relevant indices may further improve the accuracy of MASLD risk assessment. A recent study based on 3,842 participants from the U. S. National Health and Nutrition Examination Survey (NHANES) reported a positive correlation between GHR levels and the prevalence of non-alcoholic fatty liver disease (NAFLD) (OR = 1.22, 95% CI = 1.17–1.28), as well as a significant positive association between GHR and hepatic steatosis severity (*β* = 4.97, 95% CI = 4.28–5.66) (45). Importantly, this study also observed a non-linear relationship between GHR and NAFLD, consistent with the present study’s findings, suggesting that GHR is an effective predictor of fatty liver disease across diverse ethnic populations. However, it is noteworthy that previous studies have primarily focused on NAFLD, without extending to the broader category of MASLD, thereby limiting a more comprehensive assessment of metabolic factors. In addition, subgroup analyses in this study demonstrated consistent associations between GHR and MASLD across all subgroups, with a threshold effect observed in all. Furthermore, the current study validated this association specifically in non-diabetic individuals and, for the first time, clarified the mediating role of BMI—thereby significantly extending and deepening the understanding provided by prior research.

The ratio of FBG to HDL-C, an elevated GHR integrates two key metabolic abnormalities—glycemic stress (elevated FBG) and lipid dysregulation (reduced HDL-C)—and thereby promotes MASLD development through functional crosstalk between the liver and adipose tissue. First, elevated GHR is directly linked to hepatic IR. Glucotoxicity induced by hyperglycemia and the loss of HDL-C-mediated anti-inflammatory and lipid-clearing functions act synergistically to upregulate hepatic *de novo* lipogenesis (DNL) and inhibit fatty acid *β*-oxidation, initiating hepatic steatosis (46–50). Additionally, elevated GHR indicates decompensated adipose tissue lipid storage capacity, characterized by systemic obesity (reflected by increased BMI) and saturated lipid accumulation (51–53). When subcutaneous fat storage reaches saturation, excess free fatty acids (FFAs) are deposited ectopically in the liver (54, 55). This process is accompanied by adipose tissue release of proinflammatory cytokines (e.g., TNF-*α*, IL-6) and reduced expression of the anti-inflammatory adipokine adiponectin, which amplifies hepatic inflammation and oxidative stress by activating hepatic inflammatory signaling pathways, such as NF-κB and TLR4 (56–61).

The 59.86% proportion of the association, accounted for by BMI as a potential intermediary factor, suggests that the observed association between GHR and MASLD may be primarily linked through a BMI-related pathway, though causal inferences cannot be drawn. The remaining, approximately 40% of the unmediated effect, may be attributed to alternative mechanisms, such as unmeasured visceral adiposity, liver-localized IR, or direct glucolipotoxicity. Future studies should incorporate more precise assessments of body composition and molecular biomarkers to dissect these potential pathways. Notably, the present study quantified the mediating role of BMI only through statistical mediation analysis and did not explore the specific molecular targets through which GHR regulates BMI and MASLD. Thus, the underlying molecular regulatory mechanisms require further validation in subsequent basic experiments combined with clinical samples.

### 4.1 Study strengths and limitations

This study has several notable strengths. First, it was conducted using the large-sample NAGALA cohort, which is representative of the Japanese population, and included complete and reliable baseline data, ensuring the credibility of the findings. Second, a series of hierarchical statistical approaches was systematically applied to delineate the non-linear relationship and threshold effect between GHR and MASLD, and to confirm the critical mediating role of BMI. Third, subgroup analyses validated the robustness of this association across all stratified subgroups, indicating good generalizability within the study population. Fourth, the study focused on non-diabetic individuals, extending traditional NAFLD research to the broader category of MASLD with a more comprehensive metabolic definition. To the best of our knowledge, this is the first study to identify BMI as the central mediating factor in the GHR-MASLD association. Furthermore, the strict exclusion of patients with diabetes and impaired fasting glucose minimized metabolic confounding, enabling the study results to better reflect the early screening value of GHR for MASLD.

Despite these strengths, this study also has several limitations that should be acknowledged. First, as a cross-sectional study, it can only establish associations rather than causal relationships. Prospective cohort studies are therefore needed to further verify the predictive value of GHR for MASLD and clarify the causal link between them. Second, MASLD was diagnosed via abdominal ultrasonography, which has inherent limitations: it exhibits low sensitivity for mild steatosis, cannot accurately quantify hepatic fat content, nor distinguish simple steatosis from non-alcoholic steatohepatitis (NASH). Consequently, this may lead to misdiagnosis or grading bias. Third, although the statistical models were adjusted for major potential confounders, residual confounding cannot be completely ruled out due to unmeasured factors, such as dietary patterns, insulin levels, gut microbiota composition, and genetic polymorphisms. Fourth, the study population was restricted to non-diabetic individuals, which limits the generalizability of the results to other populations. Future studies should validate these findings across diverse ethnicities, regions, and clinical settings to enhance the external validity of the conclusions. Furthermore, as a cross-sectional study, the mediation analysis only quantifies the proportion of the association accounted for by BMI as a potential intermediary factor, and cannot confirm a causal mediating pathway between GHR, BMI, and MASLD; the potential intermediary role of BMI requires validation in prospective cohort studies to clarify the causal relationship.

## 5 Conclusion

In non-diabetic Japanese individuals, elevated GHR is significantly associated with increased MASLD risk, and BMI acts as a potential partial intermediary factor in this observed association. As a convenient composite biomarker from routine labs, GHR may serve as a simple, cost-effective initial screening tool for MASLD in non-diabetic adults without severe obesity or dyslipidemia.

## Data Availability

The original contributions presented in the study are included in the article/Supplementary material, further inquiries can be directed to the corresponding author.

## Glossary

MASLD: Metabolic dysfunction-associated steatotic liver disease
BMI: Body mass index
WC: Waist circumference
SBP: Systolic blood pressure
DBP: Diastolic blood pressure
ALT: Alanine aminotransferase
AST: Aspartate aminotransferase
GGT: Gamma-glutamyl transpeptidase
HbA1c: Glycosylated hemoglobin
FBG: Fasting blood glucose
TG: Triglycerides
TC: Total cholesterol
HDL-C: High-density lipoprotein cholesterol
LDL-C: Low-density lipoprotein cholesterol
GHR: Fasting blood glucose to high-density lipoprotein cholesterol ratio
OR: Odds ratios
CI: Confidence interval
***β***: Standardization regression coefficient
ROC: Receiver operating characteristic
AUC: Area under curve.
