# Supplementary material for: Fasting blood glucose to high-density lipoprotein cholesterol ratio and MASLD risk: non-linear association and BMI mediation in non-diabetic adults
Source: Front Nutr. 2026 Apr 13;13:1818931. doi: 10.3389/fnut.2026.1818931 (PMC13111242; doi:10.3389/fnut.2026.1818931)
Supplement: Supplementary file 1 [file Table_1.DOCX]

Supplementary Material

**Supplementary Tables**

**Supplemental Table 1.** The best threshold, sensitivities, specificities, and AUC of each parameter for screening MASLD in the general population and subgroup analysis for gender.

|  | **AUC** | **95% CI** | **Best threshold** | **Specificity** | **Sensitivity** |
| --- | --- | --- | --- | --- | --- |
| **General population** |  |  |  |  |  |
| GHR | 0.8149 | 0.8059-0.8239 | 3.9735 | 0.6980 | 0.8021 |
| FBG | 0.7267* | 0.7155-0.7379 | 5.1902 | 0.6140 | 0.7259 |
| HDL-C | 0.7866* | 0.7768-0.7964 | 1.2969 | 0.6895 | 0.7609 |
| **Subgroup Analysis** | | | | | |
| **Sex** | | | | | |
| \| **Men** \| \| \| \| \| \| \| --- \| --- \| --- \| --- \| --- \| --- \| \| GHR \| 0.7942 \| 0.7715-0.8168 \| 3.6903 \| 0.7922 \| 0.6735 \| \| FBG \| 0.7311* \| 0.7061-0.7561 \| 5.0237 \| 0.5870 \| 0.7609 \| \| HDL-C \| 0.7503* \| 0.7255-0.7750 \| 1.4572 \| 0.6892 \| 0.7018 \| | | | | | |
| **Women** | | | | | |
| GHR | 0.7367 | 0.7239-0.7496 | 4.4044 | 0.6289 | 0.7373 |
| FBG | 0.6489* | 0.6340-0.6638 | 5.3012 | 0.5680 | 0.6496 |
| HDL-C | 0.7094* | 0.6960-0.7227 | 1.2219 | 0.5857 | 0.7362 |
| **Age (years)**   \| **< 40** \| \| \| \| \| \| \| --- \| --- \| --- \| --- \| --- \| --- \| \| GHR \| 0.8367 \| 0.8227-0.8507 \| 3.9859 \| 0.7200 \| 0.8242 \| \| FBG \| 0.7417* \| 0.7223-0.7611 \| 5.2457 \| 0.7140 \| 0.6514 \| \| HDL-C \| 0.8094* \| 0.7939-0.8249 \| 1.2684 \| 0.7244 \| 0.7645 \|   **≥ 40**   \| GHR \| 0.8031 \| 0.7916-0.8146 \| 4.0234 \| 0.6990 \| 0.7788 \| \| --- \| --- \| --- \| --- \| --- \| --- \| \| FBG \| 0.7145* \| 0.7007-0.7284 \| 5.1902 \| 0.5802 \| 0.7404 \| \| HDL-C \| 0.7756* \| 0.7632-0.7879 \| 1.3072 \| 0.6781 \| 0.7585 \| | | | | | |
| **BMI (Kg/m^2^)** | | | | | |
| \| **< 25** \| \| \| \| \| \| \| --- \| --- \| --- \| --- \| --- \| --- \| \| GHR \| 0.8156 \| 0.8023-0.8289 \| 3.9619 \| 0.7195 \| 0.7828 \| \| FBG \| 0.7302* \| 0.7136-0.7468 \| 5.1347 \| 0.5814 \| 0.7526 \| \| HDL-C \| 0.7855* \| 0.7709-0.8000 \| 1.3408 \| 0.6752 \| 0.7677 \| | | | | | |
| \| **≥ 25** \| \| \| \| \| \| \| --- \| --- \| --- \| --- \| --- \| --- \| \| GHR \| 0.6575 \| 0.6341-0.6809 \| 4.2489 \| 0.5103 \| 0.7447 \| \| FBG \| 0.5955* \| 0.5713-0.6197 \| 5.3012 \| 0.5114 \| 0.6378 \| \| HDL-C \| 0.6409* \| 0.6172-0.6647 \| 1.2193 \| 0.5450 \| 0.6884 \| | | | | | |
| **TG (mmol/L)** | | | | | |
| **< 1.7** | | | | | |
| GHR | 0.7963 | 0.7854-0.8073 | 3.8908 | 0.6969 | 0.7729 |
| FBG | 0.7236* | 0.7104-0.7368 | 5.1902 | 0.6249 | 0.7115 |
| HDL-C | 0.7640* | 0.7521-0.7758 | 1.3512 | 0.6604 | 0.7578 |
| **≥ 1.7** |  |  |  |  |  |
| GHR | 0.6197 | 0.5871-0.6523 | 5.2919 | 0.6284 | 0.5925 |
| FBG | 0.6038* | 0.5713-0.6363 | 5.3012 | 0.5096 | 0.6505 |
| HDL-C | 0.5952* | 0.5622-0.6283 | 1.0719 | 0.5077 | 0.6599 |

* *P* < 0.01, compared with GHR. **Abbreviations:** MASLD, Metabolic dysfunction associated steatotic liver disease; GHR, Fasting blood glucose to high-density lipoprotein cholesterol ratio; BMI, Body mass index; TG, Triglycerides; CI, Confidence interval; ROC, Receiver operating characteristic; AUC, Area under the curve.

**Supplemental Table 2.** Analysis of the mediation by BMI of the associations of GHR with the risk of MASLD

|  | **Mediation effect (95% CI), *P* value** | | |  |
| --- | --- | --- | --- | --- |
|  | Total effect | Indirect effect | Direct effect | Mediation |
| BMI | 0.056 (0.048,0.064), < 0.001 | 0.033 (0.030,0.037), < 0.001 | 0.022 (0.015,0.030), < 0.001 | 59.86%, < 0.001 |

Adjust for: age, gender, hypertension, smoking status, drinking status, exercise status, HbA1c, ALT, AST, GGT, TG, and LDL-C.

**Abbreviations:** MASLD, Metabolic dysfunction associated steatotic liver disease; GHR, Fasting blood glucose to high-density lipoprotein cholesterol ratio; BMI, Body mass index; HbA1c, Glycosylated hemoglobin; ALT, Alanine aminotransferase; AST, Aspartate aminotransferase; GGT, Gamma-glutamyl transpeptidase; TG, Triglycerides; LDL-C, Low-density lipoprotein cholesterol; CI, Confidence interval.
